# Characterization of a Monoclonal Antibody Directed against *Mytilus spp* Larvae Reveals an Antigen Involved in Shell Biomineralization

**DOI:** 10.1371/journal.pone.0152210

**Published:** 2016-03-23

**Authors:** Juan Calvo-Iglesias, Daniel Pérez-Estévez, Silvia Lorenzo-Abalde, Beatriz Sánchez-Correa, María Isabel Quiroga, José M. Fuentes, África González-Fernández

**Affiliations:** 1 Immunology, Biomedical Research Center (CINBIO) and Institute of Biomedical Research of Vigo (IBIV), University of Vigo, Vigo, Spain; 2 R&D deparment, NanoImmunoTech, Vigo, Spain; 3 Veterinary Clinical Sciences, Veterinary Faculty, University of Santiago de Compostela, Lugo, Spain; 4 Centro de Investigacións Mariñas (CIMA), Consellería do Medio Rural e do Mar, Vilanova de Arousa, Spain; University of Siena, ITALY

## Abstract

The M22.8 monoclonal antibody (mAb) developed against an antigen expressed at the mussel larval and postlarval stages of *Mytilus galloprovincialis* was studied on adult samples. Antigenic characterization by Western blot showed that the antigen MSP22.8 has a restricted distribution that includes mantle edge tissue, extrapallial fluid, extrapallial fluid hemocytes, and the shell organic matrix of adult samples. Other tissues such as central mantle, gonadal tissue, digestive gland, labial palps, foot, and byssal retractor muscle did not express the antigen. Immunohistochemistry assays identified MSP22.8 in cells located in the outer fold epithelium of the mantle edge up to the pallial line. Flow cytometry analysis showed that hemocytes from the extrapallial fluid also contain the antigen intracellularly. Furthermore, hemocytes from hemolymph have the ability to internalize the antigen when exposed to a cell-free extrapallial fluid solution. Our findings indicate that hemocytes could play an important role in the biomineralization process and, as a consequence, they have been included in a model of shell formation. This is the first report concerning a protein secreted by the mantle edge into the extrapallial space and how it becomes part of the shell matrix framework in *M*. *galloprovincialis* mussels.

## Introduction

Molluscan shells are good examples of how living organisms elaborate a mineralized structure by a fully biologically controlled mineralization process called biomineralization [1–3]. The unique properties of shells as biomaterials (high fracture toughness) [4, 5] have attracted a great deal of interest and significant effort has been dedicated to the study of their structure and organic elements. Numerous opportunities are envisaged for the application of shell proteins in Nanotechnology, Bioscience and even in Biomedicine [6, 7].

Mollusc shell formation is a complex process that involves the deposition of inorganic material (95–99% CaCO_3_ in the form of calcite, aragonite or both) mixed with organic material (1–5%) [8, 9]. The organic shell matrix is only present in low quantities and it is a complex mixture of proteins, glycoproteins, chitin and acidic polysaccharides. The longitudinal section of a shell is composed of a multilayered calcium carbonate structure (usually two or three layers) covered by a external layer called the periostracum [10], which contains mostly organic material. In the aforementioned structure, *M*. *galloprovincialis* mussels have an inner nacreous layer, an outer primastic layer and an external perioustracum film covering the shell [11] in a similar way to other members of the *Mytilus* genus [12–14]. The central organ that is involved in shell formation seems to be the mantle and, in fact, the mantle edge is the most active zone for shell deposition [15]. The mantle edge in bivalves has three folds, namely the inner, middle and outer folds. Cells of the outer mantle epithelium edge zone are ultrastructurally quite different from their counterparts in the central zone [16]. Both types of cell are directly involved in mineralization through the synthesis and secretion of the array of macromolecules that self-assemble outside the cell and these macromolecules give rise to crystal formation [3]. The importance of the mantle cells in terms of protein expression is very evident, for example in the mantle tissue of the juvenile abalone *Haliotis asinine*, where the existence of 530 sequences that encode both secreted and non-secreted proteins has been demonstrated [17]. The space between the outer and the middle fold is the periostracal groove and this is the site where the periostracum is secreted. The shell, the mantle edge and the periostracum delimit the cavity called the extrapallial (EP) space. The outer fold epithelium secretes the calcifying matrix into the EP cavity. The calcifying matrix is a mixture of proteins, glycoproteins and polysaccharides that precisely self-assembles and controls the CaCO_3_ polymorphism (calcite, aragonite), the size and the shape of the crystals and, ultimately, the texture of the shell [18]. In nacro-prismatic bivalves in particular there is a different secretory regime on the outer fold epithelium. On the one hand, it has been demonstrated that the shell matrix involved in nacre deposition is secreted by cells positioned closer to the shell hinge. On the other hand, the matrix involved in controlling the prism formation is secreted by cells that are more distally positioned [19, 20]. This fact has mainly been demonstrated at the transcriptional level [21].

Extrapallial fluid (EPF) and molecules within it are believed to be involved in shell formation. Although the EP cavity is the site where the precursors for shell mineralization are supposed to concentrate and self-assemble, the EPF has not received sufficient attention [22, 23]. The EPF has been mostly characterized from the inorganic point of view [24–26], but a qualitative analysis has revealed that the EPF contains biomacromolecular materials similar to those found in the mature shell [27–29]. Nevertheless, the role of the EPF *per se* in the shell biomineralization process has been called into question[30]. Some authors defend the idea that epithelial cells of the mantle need to be in juxtaposition to the mineralizing matrix [31]. It is believed that EP proteins could participate in shell formation but they are not necessarily present in the shell [18]. Indeed, this is the situation described for two characterized EP proteins [22, 23] [32].

Previous work carried out by our group [33] led to the development of the M22.8 monoclonal antibody (mAb), which specifically detects *M*. *galloprovincialis* larvae. On using larvae of different species (*Cerastoderma edule*, *Ostrea edulis*, *Aequipecten opercularis*, *Ruditapes decussatus*, *Ruditapes philippinarum*) the antibody has shown high specificity and recognized only *M*. *galloprovincialis* and *M*. *edulis* species. This implies that the antigen recognized by M22.8 is shared by at least two species of the genus Mytilus, and this mAb has proven to be useful in the identification of mussel larvae and postlarvae [34]. Immunofluorescence and immunohistochemistry assays showed a peripheral pattern of recognition in *M*. *galloprovincialis* larvae. These results led us to suspect that M22.8 could recognize an antigen located at the mantle edge tissue and thus it could be involved in shell formation. Due to the implied significance of our hypothesis, the purpose of the study reported here was to identify the origin of the antigen recognized by the M22.8 mAb and elucidate the putative relationship between the antigen and shell formation in edible *M*. *galloprovincialis* mussels.

We show here that M22.8 recognizes an antigen (henceforth referred to as Mussel Shell Protein 22.8 or MSP22.8) not only in *Mytilus spp* larvae, but also in adult specimens. The antigen is secreted by cells of the outer fold epithelium of the mantle edge into the EPF and it finally becomes part of the shell matrix framework. The antigen is also detected inside hemocytes. Furthermore, we show here how hemocytes are able to internalize the antigen after exposure to a cell-free EPF solution. Thus, the results obtained in this study suggest a conection between the cell and shell formation.

## Materials and Methods

### Ethics Statement

The Mediterranean mussels (*M*. *galloprovincialis*) used in this study, which were common and not endangered invertebrate species, were purchased from a local seafood market (Flipper fish shop, Mercado do Berbés, Vigo, Spain). Permits were not required for the study, which complied with all relevant regulations and all efforts were made to minimize possible animal suffering.

### Mussels

Mussel larvae were obtained by the induction of spawning of mature individuals of *M*. *galloprovincialis*, taken from culture rafts in the Galician Rías, according to previously described methods [33]. Two day-old larvae and 16 day-old larvae were conserved in cryovials (Nunc, Brand Products, Roskilde, Germany) in phosphate-buffered saline (PBS), pH 7.4 (0.15 M NaCl, 2.7 mM KCl, 1 mM Na_2_HPO_4_, and 1.8 mM KH_2_PO_4_) and in dimethyl sufoxide (DMSO, Sigma, St. Louis, USA) 10% at –80°C or in liquid nitrogen.

Adult specimens of mussels (M. galloprovincialis) purchased from a local seafood market were collected from aquaculture populations of Galician bays, ría de Vigo (42°15′N 8°45′O) and ría de Vilagarcia de Arousa (42°30′00″N 8°56′00″O).

### Adult tissues and larvae lysates

*M*. *galloprovincialis* adult individuals were dissected with a scalpel blade to obtain fragments of the main macroscopical tissues (byssal retractor muscle, mantle, mantle-gonadal tissue, gills, digestive gland, foot and labial palps). Fragments were transferred to a 2 mL sterile centrifuge tube, kept on ice and lysed in a lysis buffer (Tris-HCl 10 mM, pH 8, NaCl 150 mM, EDTA 2.5 mM and 1% NP-40). A protease and phosphatase inhibitor cocktail was added (Complete Mini and PhosphoStop from Roche Ltd., Basel, Switzerland). In the case of larvae, frozen samples were defrosted, washed with sodium phosphate buffer (PBS) and ultrapure water and lysed with the same lysis buffer as described for adult tissues. A sonication step was also carried out.

### Extraction and fractionation of extrapallial fluid and hemolymph

Extrapallial fluid (EPF) from *M*. *galloprovincialis* adult mussels was extracted by inserting a needle into the extrapallial space and removing the fluid into a sterile syringe. Punction was carried out carefully in order to avoid contact with the mantle surface and to avoid contamination with water or tissue debris. EPF was pooled, filtered through 0.45 μm and 0.22 μm filters, and immediately transferred to 1.5 mL sterile centrifuge tubes and kept on ice. Centrifugation was performed at 16,000 g for 10 minutes at 4°C. The supernatant was retained and kept on ice in cases where it was used immediately or was otherwise frozen at –20°C. Hemolymph was extracted by inserting a needle into the adductor muscle in *M*. *galloprovincialis* adult mussels. Samples of hemolymph were treated in a similar way to that described for EPF.

### Shell material

Adult shells of *M*. *galloprovincialis* were extensively brushed to remove epibionts and soaked in 3% NaOCl for one hour, rinsed with distilled water and dried. This treatment allowed partial removal of the periostracum. Clean shells were crushed into a powder. Shell powder was completely demineralized with 0.5 M EDTA (pH 7.8) in a dialysis cassette (Slide-A-Lyzer® Dialysis Cassette, Thermo Scientific, USA). After demineralization, EDTA-soluble fractions were dialyzed against sodium phosphate buffer (PBS) and desalted with a centrifugal filter (Amicon Ultra-0.5 Centrifugal Filter, Merck Millipore, Germany). EDTA-insoluble fractions were extensively washed with ultrapure water. The two EDTA fractions were lyophilized and stored at –20°C prior to use. EDTA-soluble fractions were rehydrated with Laemmli sample buffer (Bio-Rad Laboratories, Inc., USA). Insoluble lyophilized samples were rehydrated with lysis buffer (urea 7 M, thiourea 2 M, CHAPS 4% w/v, dithiothreitol 3% w/v). The rehydrated samples were further precipitated with a commercial kit to remove contaminants (ReadyPrep™ 2-D Cleanup Kit, Bio-Rad Laboratories, Inc., USA). Protein pellets were finally resuspended by adding an appropriate volume of Laemmli sample buffer.

### Protein electrophoresis

Electrophoresis experiments were carried out using a Mini-PROTEAN^®^ 3 or a Mini-PROTEAN^®^ Tetra cell electrophoresis unit (Bio-Rad Laboratories, Inc., USA). The protocols (SDS-PAGE) were carried out according to the manufacturer’s instructions. Reagents of electrophoresis grade were obtained from Bio-Rad Laboratories Inc., electrophoresis grade ammonium persulfate was purchased from Sigma Aldrich and 1,2-bis(dimethylamino)ethane was obtained from Merck Millipore. A broad range of molecular weight markers were purchased from Bio-rad Laboratories Inc. (Precision Plus Protein™ Dual Color Standards) and Thermo Scientific (Scientific PageRuler Plus Prestained Protein Ladder). Unless stated otherwise, samples were prepared following the Laemmli protocol [35]. Dithiothreitol (DTT, Bio-rad Laboratories Inc.) was selected as the reducing agent. DTT was added to a final 1x concentration of 50 mM. Samples were run on 10% SDS-PAGE vertical minigels under both non-reducing/reducing conditions (DTT included). Electrophoretic conditions were 200 V at constant current according to the manufacturer’s instructions. Minigels were stained with Coomassie blue or silver.

### Western blot

Western blot assays [36] were carried out using minigels [37] transferred to PVDF membranes (Immun-Blot^®^, BioRad Laboratories Inc.) using a Trans-Blot^®^ Turbo™ Transfer System (BioRad Laboratories Inc.). Transfer conditions were 25 V during 30 minutes. Membranes were washed in Tris-buffered saline with 1% Tween 20 (TBST) and blocked with 5% skimmed milk (Sigma-Aldrich Co.) in TBST overnight. After blocking, membranes were washed 3 times with TBST and incubated for 2 hours with M22.8 hybridoma supernatant (1:10 dilution in TBST with 2,5% skimmed milk) at room temperature (RT). For colorimetric Western blotting assays, goat anti-mouse IgG antibodies conjugated to AP (Dako) diluted 1:1,000 in TBST with 2.5% skimmed milk were used as secondary antibodies (1.5 hours, RT). Goat anti-mouse IgG antibodies conjugated to horse rabbit peroxidase (HRP) (Dako) diluted 1:50,000 in TBST with 2.5% skimmed milk were used as secondary antibodies (1 hour, RT) for chemiluminescent Western blot assays. Colorimetric Western blot assays were revealed with 1-Step NBT/BCIP (Thermo Scientific) whereas the Immun-Star™ WesternC™ Chemiluminescent Kit (BioRad Laboratories Inc.) was selected for chemiluminescent assays. Protein bands were analyzed using the ChemiDoc XRS imaging system in conjunction with ImageLab software (BioRad Laboratories Inc.).

### Immunohistochemistry

Mussels were opened with a razor blade by cutting the adductor muscle and the whole body was immediately immersed in Bouin solution (picric acid 75 mL, 40% formol 20 mL and glacial acetic acid 5 mL), dehydrated in graded ethanol solutions and embedded in paraffin according to standard hystological procedures. Sections (3 μm thick) were cut with a rotary microtome and mounted on silanized slides. Sections were deparaffinized in xylene, rinsed in acetone, immersed in distilled water and washed in PBS. Endogenous peroxidase activity was quenched by incubating sections in blocking solution (ChemMate Peroxidase Blocking solution, Dako) for 30 minutes. Sections were then washed in phosphate buffer with Tween (PBST) and incubated with the M22.8 mAb (diluted 1/100 in Dako Real Antibody diluents) for 2 hours at RT. The sections were briefly rinsed in PBST (× 5 min) and were incubated for 30 minutes with HRP-rabbit/mouse antibodies (Dako Real Envision/HRP, Rabbit/Mouse). After several rinses in PBST, immunocomplexes were visualized with Vector^®^ VIP (Vector Laboratories) or DAB solution (Dako Real DAB+Chromogen and Dako Real Substrate buffer). Reaction was stopped with distilled water. Finally, sections were counterstained with haematoxylin for 20 seconds and mounted. Control sections were incubated in the same way, except for the incubation with M22.8, where antibody diluent (Dako) was used instead.

### Flow cytometry with hemocytes from hemolymph and extrapallial fluid

Fresh hemolymph and EPFs were carefully extracted as described before. Samples were filtered through a 100 μm nylon mesh, transferred into micro-tubes and kept on ice to minimize cell clumping, in a similar way to that described for hemolymph collection in Suminoe oyster [38]. Filtered samples were short centrifuged at 16,000 g, 13 seconds, 4°C. Cell pellets containing hemocytes were further washed with filtered clean sea water to remove debris. Flow cytometry assays were perfomed on both intact and fixed-permeabilized hemocytes. Acetone was selected as the Fixation-Permeabilization Solution (10 min at –20°C). Cell pellets were incubated overnight at 4°C with 100 μL of M22.8 hybridoma supernatant. To avoid non-specific binding, a blocking agent (FcR Blocking agent, Immunostep) was added for 10 minutes. Goat anti-mouse IgG (H+L) antibodies coupled to fluorescein isothiocyanate (FITC, Southern Biotech, USA) were used as secondary antibodies (30 min, 4°C). Unstained cells, i.e., samples not incubated or only incubated with secondary antibodies, were included as control cells. Samples were analyzed in a BD Accuri™ C6 Flow Cytometer (BD Biosciences) and the data were analyzed using BD Accuri C6 Software (eBiosciences).

### Endocytosis assay

Fresh EPF was centrifuged to separate hemocytes from EPF supernatant. The EPF supernatant was filtered (0.45 and 0.22 μm) and kept as a cell-free EPF solution. Fresh hemolymph individually extracted from 4 mussels was short centrifuged at 16,000 g, 13 seconds, 4°C. Hemocytes were washed with filtered clean sea water, resuspended and incubated in the presence of cell-free EPF solution for 2 hours at 15°C in the absence of light. Clean sea water was used instead of cell-free EPF solution to provide a negative control. Hemocytes were assayed by flow cytometry as described above.

## Results

### M22.8 mAb recognizes the MSP22.8 antigen in the mantle edge of adult *M*. *galloprovincialis* mussels

**Fig 1:**
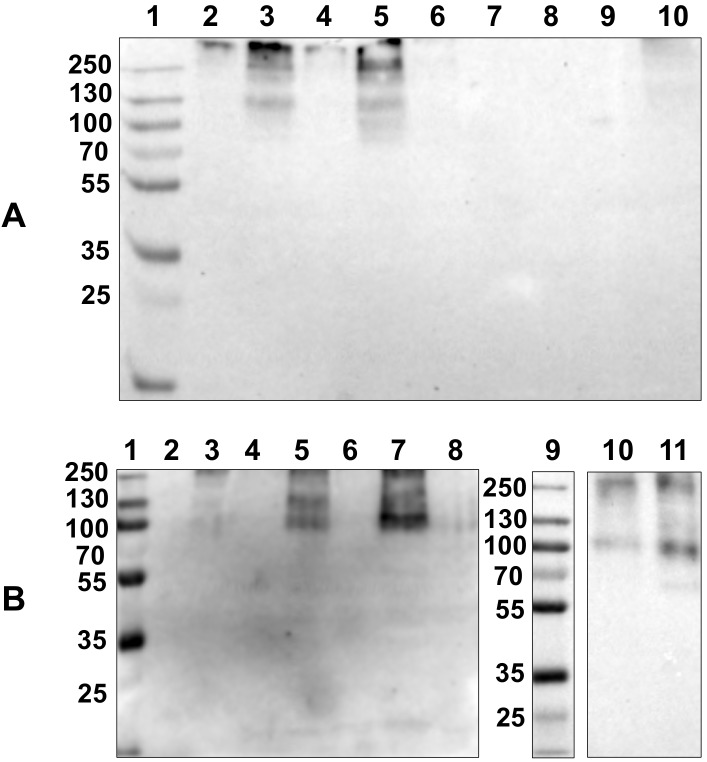
Chemiluminescent Western blot of lysates of several tissues from adult *M*. *galloprovincialis* mussels and comparison with larvae lysate. SDS-PAGE 10% under non-reducing (**A**) conditions: (1) molecular weight standards (kDa); (2,4,6,9 and 10) central mantle; (3, 5) mantle edge; (7) digestive gland and (8) foot. SDS-PAGE 10% under reducing (**B**) conditions: (1,9) molecular weight standards; (2) empty well; (3,4,6,8) central mantle, (5,7) mantle edge; (10) 2 day-old larval lysate and (11) 16 day-old larval lysate.

Major tissues of adult *M*. *galloprovincialis* mussels were tested by Western blot. In particular, lysates of the central mantle, mantle edge, digestive gland, labial palp, foot and byssal retractor muscle were evaluated. Several experiments were conducted under both reducing and non-reducing conditions (Fig 1).

The M22.8 mAb recognizes the MSP22.8 antigen only in tissue lysates from the mantle edge. The central mantle, foot and digestive gland do not express the antigen (Fig 1A) and other tissues tested, such as byssal retractor muscle and labial palp, were also negative (S1 Fig). Under non-reducing conditions, the antigen appears in the mantle edge as a main band at approximately 130 kDa (Fig 1A), although an upper strong positive area is also visible (> 250 kDa). Under reducing conditions (Fig 1B) the protein band at 130 kDa has a reduced intensity or almost disappears completely and it coexists with a more intense band at approximately 100 kDa.

As shown in Fig 1B (lanes 10 and 11), a band at 100 kDa is also observed for 2 day-old larvae and 16 day-old larvae, indicating that this antigen is expressed at very early stages of development and is maintained throughout the life of the mussel.

### Immunohistochemistry: the MSP22.8 antigen is expressed on the outer lobe epithelium

**Fig 2:**
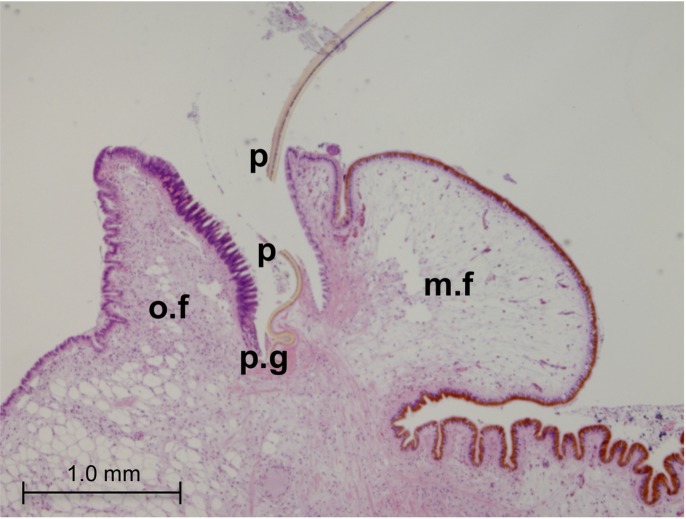
Histology of the mantle edge in adult *M*. *galloprovincialis*. Hematoxilin-eosin stain, 4× microphotographs. (o.f): outer fold; (m.f): middle fold; (p.g): periostracal groove; (p): periostracum.

Tissue sections from the mantle edge of adult *M*. *galloprovincialis* were first stained with haematoxylin-eosin (Fig 2). The mantle edge in *Mytilus spp* has three folds (or lobes), namely the outer, middle and inner folds. The space between the outer and middle folds is of particular interest since it constitutes the periostracal groove. At the outer fold, the outer epithelium faces the inner shell surface while the inner epithelium faces the periostracal groove.

**Fig 3:**
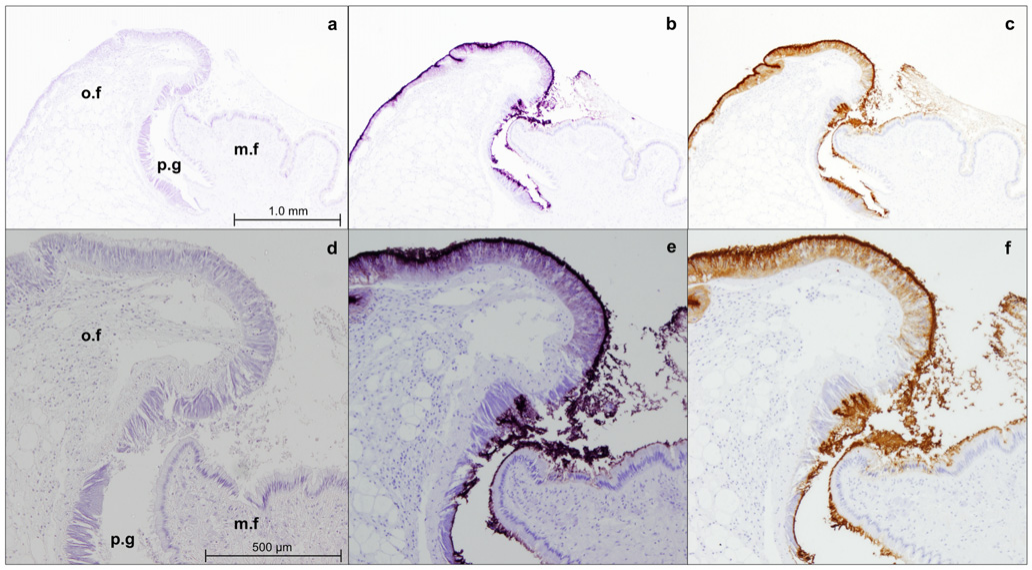
Immunostaining of the mantle edge of adult M. galloprovincialis with M22.8 mAb. Microphotographs were taken at 4× (a, b, c) and 10× (d,e,f). (o.f): outer fold; (m.f): middle fold; (p.g): periostracal groove; (a,d) negative control; (b,e): immunostaining revealed with VectorVip; (c,f) immunostaining revealed with DAB.

Immunohistochemistry staining with M22.8 shows that the mAb recognizes the antigen only on the outer lobe epithelium in the mantle edge sections (Fig 3) and is negative for the rest of the tissues tested, such as digestive gland, foot, gills, gonadal tissue (S2 Fig).

As shown in Fig 3E and 3F, epithelia stained positively exhibit a granular pattern.

**Fig 4:**
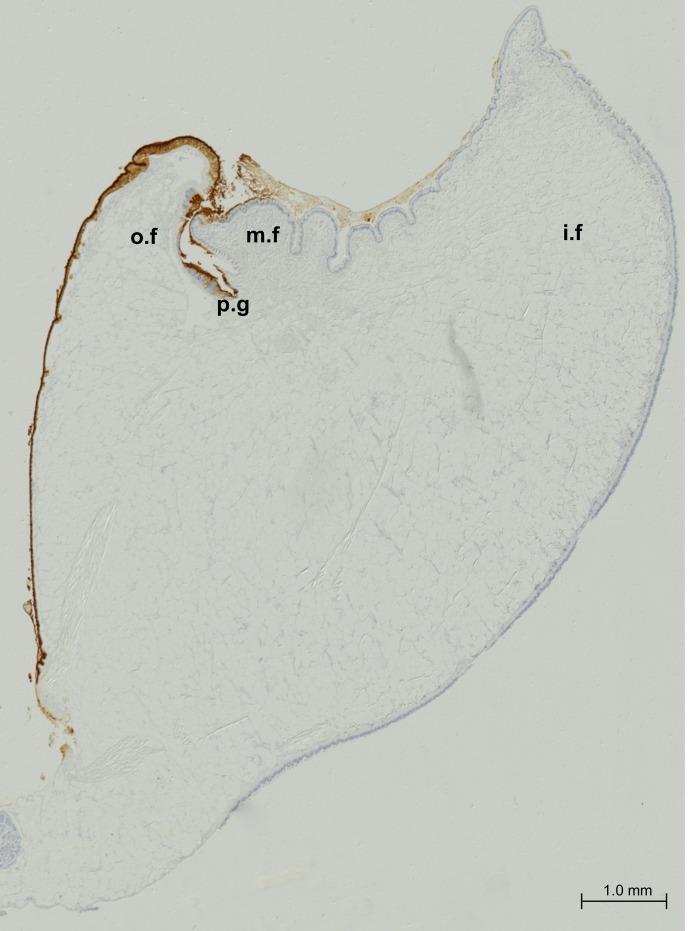
Immunostaining of the mantle edge in adult *M*. *Galloprovincialis* with M22.8 mAb and revealed with DAB. (o.f): outer fold; (m.f): middle fold; (p.g): periostracal groove; (i.f): inner fold.

A set of microphotographs at 5× were taken to cover the whole mantle edge of *M*. *Galloprovincialis* (see Fig 4). Microphotographs were automatically mounted for illustrative purposes. It can be observed that recognition of the M22.8 mAb is restricted to the epithelium of the outer fold until its attachment to the shell (pallial line), with a negative result obtained for the rest of the epithelia.

### Detection of the MSP22.8 antigen in the extrapallial fluid (EPF) but not in the hemolymph

**Fig 5:**
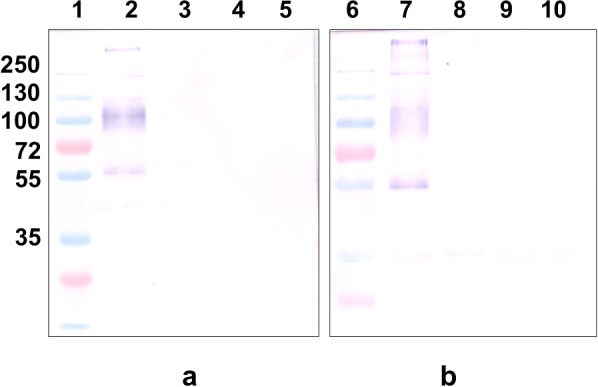
**Western blot assays on EPF and hemolymph from adult *M*. *galloprovincialis* mussels under reducing (a) and non reducing (b) conditions.** (1, 6) Molecular weight markers (kDa); (2, 7) pool of EPFs; (3, 8; 4, 9; 5, 10) hemolymph from mussel 1, 2 and 3, respectively.

A pool of EPFs from several mussels and individual hemolymph samples from adult *M*. *galloprovincialis* mussels were assayed by Western blot under reducing and non-reducing conditions (Fig 5). The MSP22.8 antigen was found to be strongly positive in the extrapallial fluid under both sets of conditions. In contrast, the hemolymph samples were negative to this antigen.

The antigen recognized by the M22.8 mAb shows two bands in the extrapallial fluid: a sharp and well-defined band at 55 kDa and a more diffuse band at 100 kDa. A third and less intense band was also observed in the range 130–250 kDa and this suggests the presence of dimeric or multimeric forms of the antigen. Major differences were not observed on comparing reducing and non-reducing conditions, since the bands described above appear in both cases with similar intensities.

### Detection of the MSP22.8 antigen in EP hemocytes by Western blot

**Fig 6:**
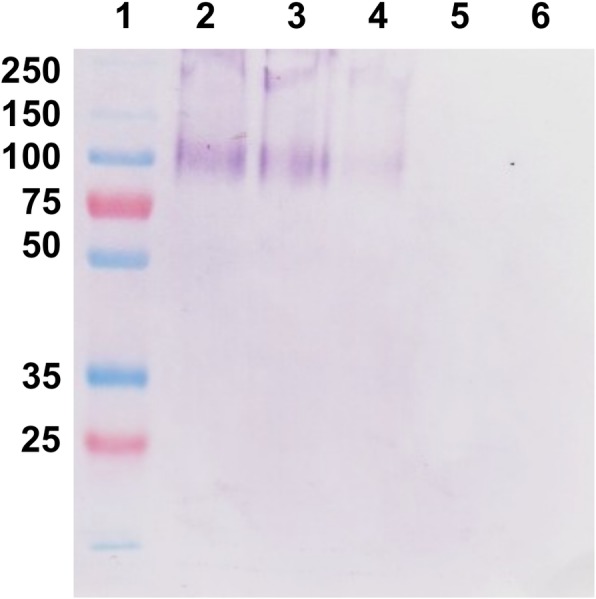
Western blot assays on hemocytes from adult *M*. *galloprovincialis*. (1) molecular weight markers (kDa); (2, 3, 4) EP hemocytes; (5) hemolymph hemocytes; (6) empty well.

It was desirable to test if the antigen was also present in the hemocytes. Hemocytes from EPF and hemolymph were extracted and assayed to investigate the presence of the antigen recognized by M22.8 mAb (Fig 6). It was found that the mAb recognizes a band at 100 kDa in EP hemocytes. However, a signal was not observed for samples containing hemolymph hemocytes, which indicates that there are differences in the cells depending on the fluid in which they are located, i.e., EPF or hemolymph.

### EP hemocytes, but not hemolymph hemocytes, show intracellular staining with M22.8 mAb

**Fig 7:**
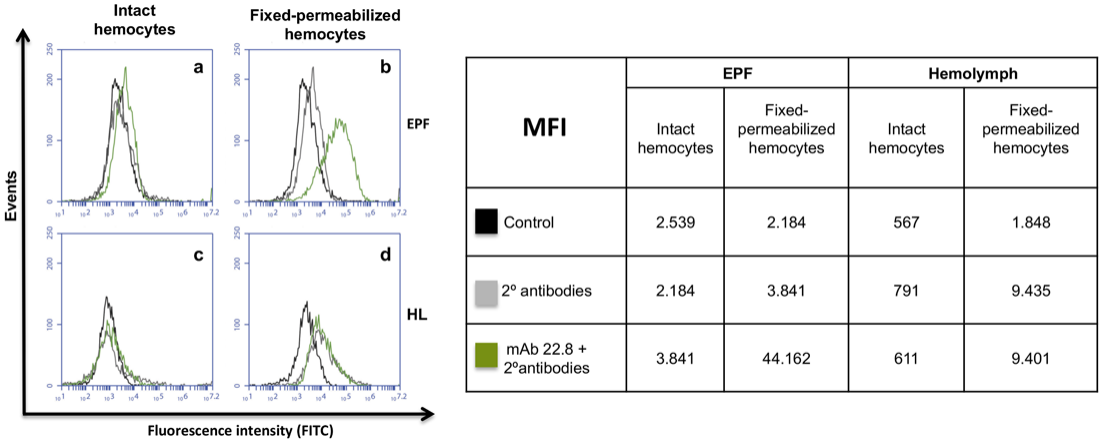
**Flow cytometry analysis of intact and fixed-permeabilized EPF (a,b) and hemolymph (c,d) hemocytes**. Hemocytes were incubated with M22.8 mAb followed by FITC-labelled secondary antibodies. (MFI): median fluorescence intensity; (HL): hemolymph.

Fresh EPF and hemolymph hemocytes were further assayed by flow cytometry. As indicated in Fig 7, intact hemocytes, either from EPF(a) or hemolymph (c), on the cell surface showed very low or zero staining with M22.8 mAb. A different situation was found in permeabilized EP hemocytes (b), which were clearly stained, indicating that in these hemocytes the MSP22.8 antigen is located intracellularly. In contrast, the staining on permeabilized hemolymph hemocytes (d) was negative and the results were similar to those found with non-permeabilized intact cells or with permeabilized cells incubated only with secondary antibodies.

**Fig 8:**
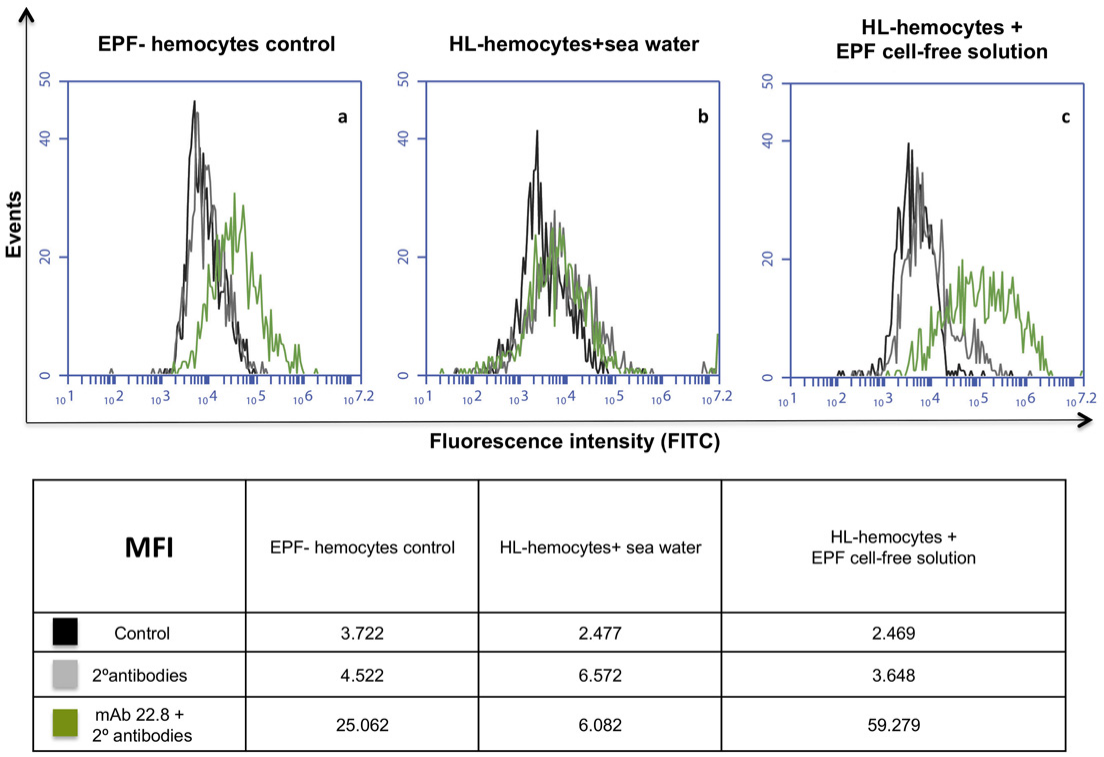
Flow cytometry analysis of permeabilized hemocytes. (a) EP hemocytes used as positive control; (b) hemolymph hemocytes exposed to sea water; (c) hemolymph hemocytes exposed to EPF cell-free solution. Cells were incubated with the M22.8 mAb followed by FITC-labelled secondary antibodies. (MFI): median fluorescence intensity; (HL): hemolymph.

In order to ascertain whether intact hemolymph hemocytes could internalize the antigen present in the EPF media, these hemocytes were exposed to an EPF cell-free solution and then fixed and permeabilized. As can be seen in Fig 8, hemolymph hemocytes exposed to EPF become positive and this suggests the uptake of the antigen present in the EPF solution.

### Detection of the MSP22.8 antigen in the shell organic matrix

**Fig 9:**
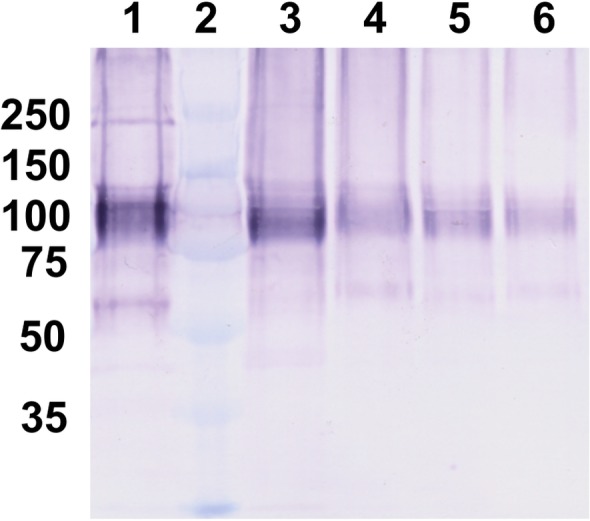
Western blot assay on M. *galloprovincialis* shell organic matrix. (1) EPF as positive control; (2) molecular weight markers (kDa); (3,4,5,6) *Mytilus galloprovincialis* shell organic matrix from different mussels.

The presence of the antigen in the mantle edge, EPF and EP hemocytes led us to postulate that the antigen could be transported to the mussel shell and form part of the shell organic matrix. In an effort to clarify this issue, EDTA-insoluble fractions obtained from *M*. *galloprovincialis* shell organic matrix were assayed by Western blot. It can be observed in Fig 9 that the M22.8 mAb recognizes the antigen in the mussel shell (bands at 100 kDa and 55 kDa) in a similar way to that found for the EPF samples, thus indicating that the antigen is located in the shell organic matrix.

## Discussion

In the present work it has been demostrated that M22.8 mAb recognizes an antigen (MSP22.8) not only in *M*. *galloprovincialis* larvae but also in adult specimens, suggesting that it is expressed at all stages of mussel life. Indeed, our results show that MSP22.8 is detected in the mantle edge, EPF (including EP hemocytes) and shell organic matrix and that it forms part of the matrix framework.

Electrophoretically, MSP22.8 shows different behaviour depending on the samples considered and the conditions assayed. The antigen extracted from the mantle edge appears in Western blot assays as a main band at approximately 130 kDa under non-reducing conditions. When DTT is added to the loading buffer, the molecular weight of MSP22.8 shifts to approximately 100 kDa, suggesting that the 130 kDa protein could be composed of 2 subunits bound by a disulfide bond. However, in the case of the EPF samples, M22.8 recognizes two bands, namely a diffuse band at approximately 100 kDa and a sharper one at approximately 55 kDa, and major differences were not observed between reducing or non-reducing conditions.

A possible explanation for these differences in the size of the MSP22.8 antigen from the adult mantle edge and the EPF could be due to the production of a pre-protein in the mantle, which could be processed further. This pre-protein could perhaps be produced as a heterodimeric protein. It has been reported that in *M*. *galloprovincialis* the novel blue mussel shell protein (BMSP) is produced as a pre-protein consisting of a signal peptide and two proteins, BMSP 120 and BMSP 100 [39]. MSP22.8 could be processed in a similar way and this would generate two proteins after cleavage (100 kDa and 55 kDa), which would explain the results obtained in the Western blot assays.

The diffuse appearance of the 100 kDa band could be due to post-translational modifications such as phosphorylation and glycosylation. This finding is very common in proteins associated with mineralized tissues [3], including shell proteins in molluscs [40, 41]. It is important to note that MSP22.8 is also detected in *M*. *galloprovincialis* shell extracts and shows similar bands (100 kDa and 55 kDa) to those found in the EPF, thus suggesting that the protein is processed in the EPF before reaching the shell.

Previous results obtained with M22.8 mAb using indirect immunofluorescence and immunohistochemistry techniques showed a peripheral recognition pattern in *Mytilus galloprovincialis* early larvae and post-larvae (2–34 days old) [33, 42]. The results reported in this study, however, confirm the presence of the MSP22.8 antigen in *M*. *galloprovincialis* larvae lysates. The MSP22.8 antigen is expressed at very early stages (2-day old larvae) and this coincides with the formation of the early shell, the prodissoconch [43]. Furthermore, according to our results this antigen seems to be expressed throughout the whole life of the mussel.

Another interesting question concerns the immunolocalization of MSP22.8 in living tissues. According to our results, MSP22.8 is secreted by cells of the mantle edge epithelium. In particular, cells of the outer epithelium from the apical zone of the outer lobe up to the pallial line (the place where pallial muscle is attached to the shell). The region where MSP22.8 is expressed coincides with the region described for the expression of shell proteins belonging to the prismatic layer in nacro-prismatic bivalves [19, 21]. In particular, this situation has been demostrated for some shell proteins such as the pearl oyster *Pinctada fucata*, MSI31 [20] and Aspein [44]. Furthermore, it seems that there is a clear limit between the nacre-secreting and the prism-secreting cells [45]. Although we do not have evidence that MSP22.8 is located within the shell layers, we presume that MSP22.8 could be involved in the formation of the prismatic layer.

MSP22.8 is detected both in EPF and hemocyte lysates extracted from the extrapallial cavity. In contrast, neither hemocytes from hemolymph nor hemolymph itself are positive in the Western blot assay. This finding can be explained if we assume that the mantle epithelium is impermeable to medium-high molecular weight molecules. In the quahog *Mercenaria mercenaria*, an experiment with bovine serum albumin (66 kDa) showed that the outer mantle epithelium forms an effective barrier to at least some high molecular weight proteins [46]. Similar results were previously reported for the freshwater snail *Biomphalaria glabrata* in relation with horseradish peroxidase (40 kDa) [47]. Given its molecular weight, flux within surrounding tissue or hemolymph should not occur with MSP22.8 after its secretion by the mantle edge. This would explain why neither hemolymph nor hemocytes in these regions are positive.

With the aim of confirming the presence of MSP22.8 in EP hemocytes and to obtain information about the role of these immune cells, we designed flow cytometry assays that included an endocytosis experiment in which hemocytes from hemolymph were exposed to a cell-free EPF. The results confirmed that permeabilized EP hemocytes were strongly positive and this indicates that MSP22.8 is present within these EP cells. In contrast, permeabilized hemolymph hemocytes were negative to staining with the antibody. However, surprisingly the presence of MSP22.8 dramatically increased on these hemolymph hemocytes after exposure to a cell-free EPF (containing the MSP22.8 antigen), indicating that MSP22.8 can, in fact, be internalized by these cells.

On the basis of the results outlined above, we suggest that MSP22.8 is secreted by mantle cells into the EPF and is then internalized by hemocytes therein, which transport the antigen to the shell. Apart from their functions as the cells responsible for innate immunity in bivalves [48], the role that the EP hemocytes play in shell formation is still unclear. It is believed that these hemocytes play an important role in the biomineralization process [45], although this possibility has not been confirmed. Some data on the oyster *Crassostrea virginica* reveal that these immune cells are able to carry calcite to the mineralization site [49]. Additionally, a 48 kDa phosphoprotein previously described as a component in the shell matrix of *Crassostrea virginica* was also detected in hemocytes from these species [50].

In the classical view of shell formation, the normal shell mineralization phenomenon is seen as a succession of compartments [10, 16]. The mantle organ, periostracum and extrapallial cavity are considered to be the three pillars. The mantle would be the central organ, in particular the outer mantle ephitelium, which secretes the array of molecules that participate directly in the crystal formation [3]. According to the classical approach, outer epithelium would control the mineralization process remotely since there is no direct contact between the outer epithelium and the shell mineralization zone. Molecules secreted into the extrapallial cavity would be self-assembled and they reach the shell growing area without the participation of any cell [45].

However, this classical theory has been called into question by several authors [7, 18, 45]. In particular, if one focuses on the nacre formation [30], it seems that mantle cells are in close contact with the mineralization front. Prism and nacre would be assembled from different protein repertoires, thus suggesting the existence of a cell secreting differentiation [51]. A new model has been proposed for oysters [52], where the shell matrix protein production could not be restricted to the mantle organ and mantle proteins could be transported to the shell by a specific type of hemocyte. The complexity of shell formation and the involvement of hemocytes and exosomes have been described in the Pacific oyster Crassostrea gigas [53]. The diversity of protein domains suggests that the role of the shell matrix proteins may be broader than expected and not restricted to crystallization processes [54].

Biomineralization of the mollusc shell is far from being well understood. Following the path of a protein after it is secreted is not sufficient to understand fully the complex phenomenon of shell formation. However, the results described here could help to shed some light on the importance of the mantle-secreted protein, EPF and hemocytes in the shell biomineralization process. We suggest that the calcifying matrix could be cell-guided to the mineralization front. Cellular control of mineralization would be the responsibility of both the mantle cells and the hemocytes. Due to their mobility within the extrapallial cavity, hemocytes could reach parts of the shell that cannot be reached by the mantle cells.

**Fig 10:**
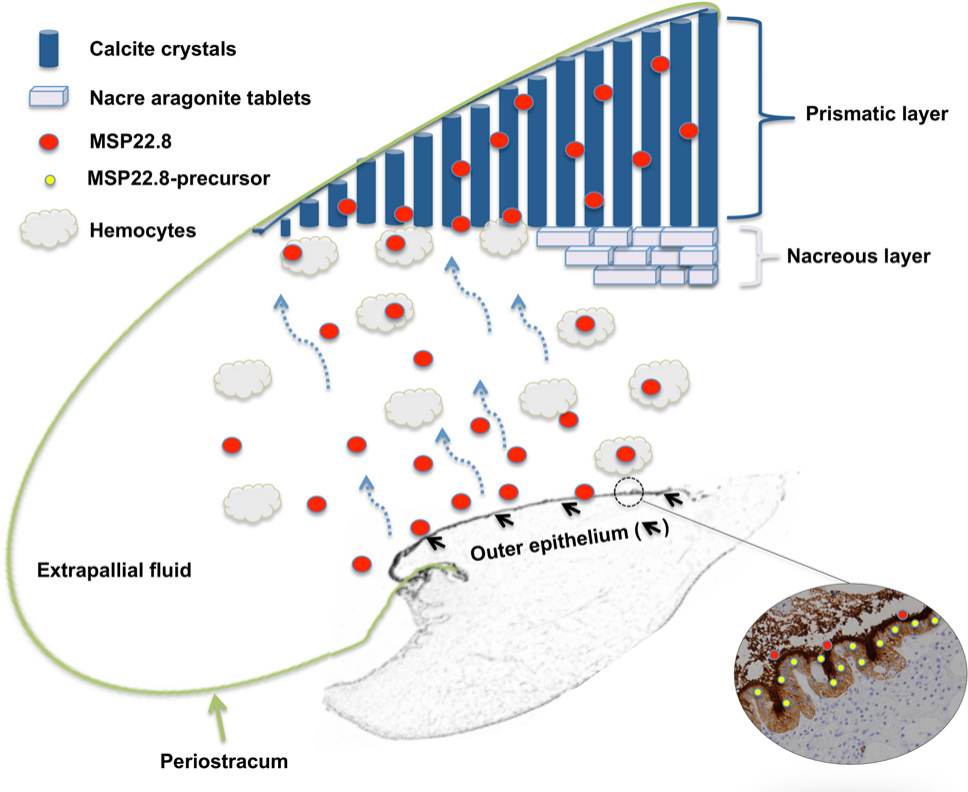
Model of the cell-mediated shell formation in *M*.*galloprovincialis*. The size of the extrapallial cavity is enlarged for the sake of clarity.

Based on the fact that MSP22.8 is expressed in the mantle edge, our hypothesis starts with the production of the antigen. MSP22.8 seems to be secreted as a pre-protein in the mantle edge epithelia and then further processed in the EPF medium. In that medium, the antigen could be transported by the EP hemocytes to the mineralization front, where it could form part of the shell mineralization matrix, particularly within the prismatic layer (Fig 10).

## Supporting Information

S1 Fig Western blot assays on adult tissues of *Mytilus galloprovincialis*. SDS-PAGE 8% under non-reducing conditions: (1) molecular weight markers (kDa); (2, 3) mantle edge; (4) labial palp; (5) byssal retractor muscle.
(PDF)

S2 Fig Immunostaining of adult tissues of *M*. *Galloprovincialis*. Microphotographs were taken at 10×.Samples of gonadal tissue (a,b), gills (c,d), foot (e, f) and digestive gland (g, h) were incubated with antibody diluent (a, c, e,g) or M22.8 (b,d,f,h) and revealed with DAB.
(PDF)
