# Supplementary material for: Characterization of a Monoclonal Antibody Directed against Mytilus spp Larvae Reveals an Antigen Involved in Shell Biomineralization
Source: PLoS One. 2016 Mar 23;11(3):e0152210. doi: 10.1371/journal.pone.0152210 (PMC4805170; doi:10.1371/journal.pone.0152210)
Supplement: S1 Fig — SDS-PAGE 8% under non-reducing conditions: (1) molecular weight markers (kDa); (2, 3) mantle edge; (4) labial palp; (5) byssal retractor muscle. (PDF) [file pone.0152210.s001.pdf]

Supporting information

Calvo-Iglesias et al.

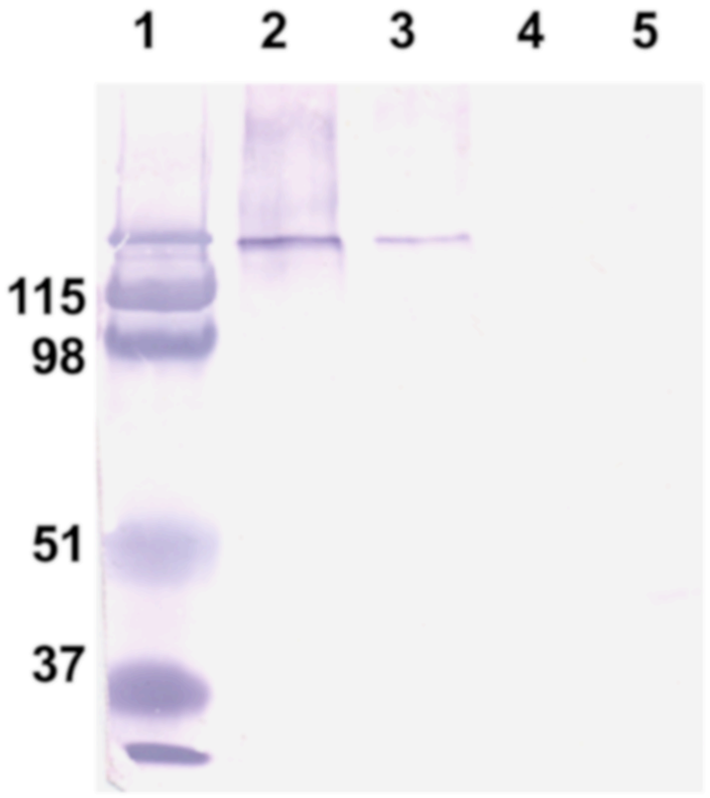

S1 Fig. Western blot assays on adult tissues of *Mytilus galloprovincialis*.

SDS-PAGE 8% under non-reducing conditions. (1) molecular weight markers (kDa); (2, 3) mantle edge ; (4) labial palp; (5) byssal retractor muscle.
