# Supplementary material for: Characterization of a Monoclonal Antibody Directed against Mytilus spp Larvae Reveals an Antigen Involved in Shell Biomineralization
Source: PLoS One. 2016 Mar 23;11(3):e0152210. doi: 10.1371/journal.pone.0152210 (PMC4805170; doi:10.1371/journal.pone.0152210)
Supplement: S2 Fig — Microphotographs were taken at 10×.Samples of gonadal tissue (a,b), gills (c,d), foot (e, f) and digestive gland (g, h) were incubated with antibody diluent (a, c, e,g) or M22.8 (b,d,f,h) and revealed with DAB. (PDF) [file pone.0152210.s002.pdf]

Supporting information

Calvo-Iglesias et al.

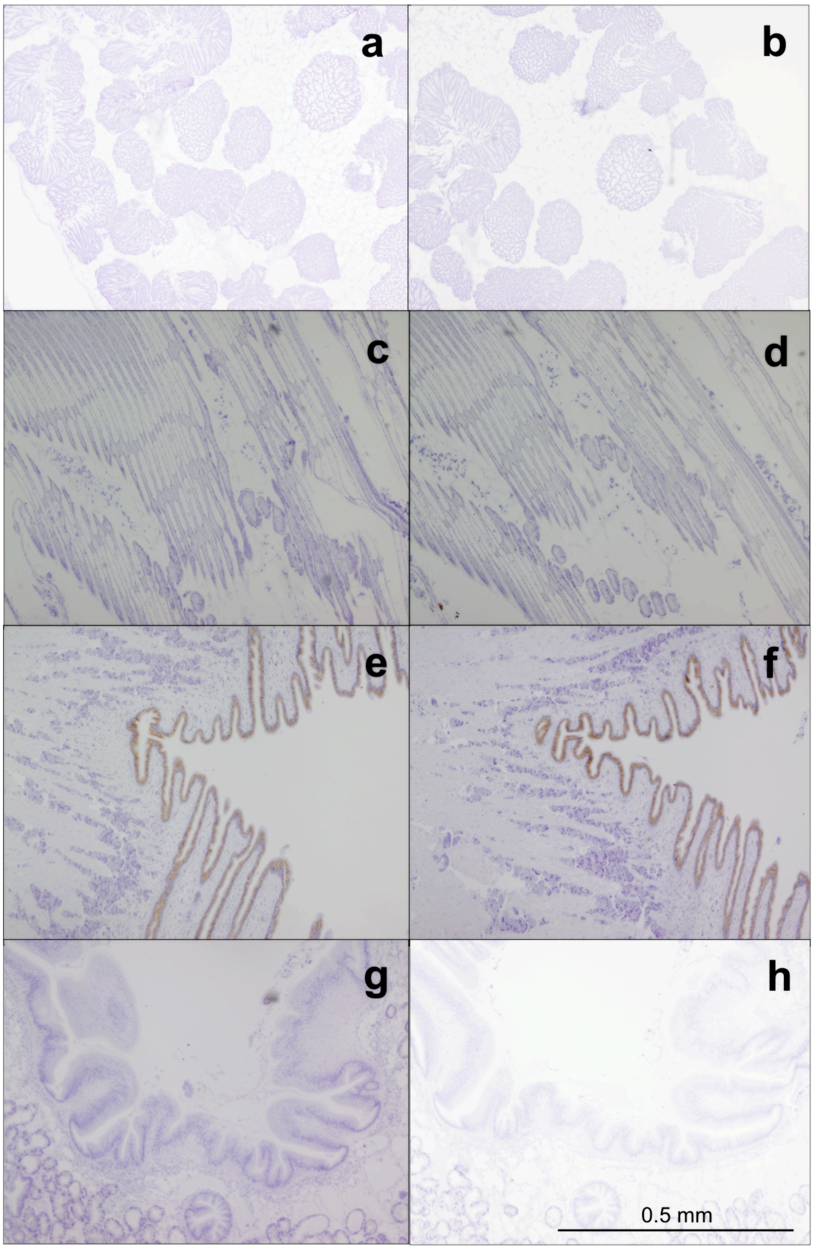

S2 Fig. Immunostaining of adult tissues of *M. Galloprovincialis*. Microphotographs were taken at 10× .Samples of gonadal tissue (a,b), gills (c,d), foot (e, f) and digestive gland (g, h) were incubated with antibody diluent (a, c, e,g) or M22.8 (b,d,f,h) and revealed with DAB.
